# The amalgamation of cellular metabolism and immunology for host immunity

**DOI:** 10.1002/cti2.1123

**Published:** 2020-03-11

**Authors:** Justine D Mintern, Katrina J Binger

**Affiliations:** ^1^ Department of Biochemistry and Molecular Biology Bio21 Molecular Science and Biotechnology Institute, The University of Melbourne Parkville VIC Australia; ^2^ Department of Biochemistry and Genetics La Trobe Institute for Molecular Science, La Trobe University Bundoora VIC Australia

## Abstract

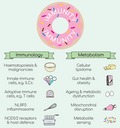

Cellular metabolism is a fundamental mechanism to provide all cells with energy in the form of adenosine triphosphate (ATP). The study of metabolic pathways, however, reawakens horror memories to many an immunologist – of long undergraduate lectures describing complicated, interconnected pathways of countless enzymatic reactions, regulatory mechanisms and metabolites. Yet as cellular metabolism has emerged as a critical modulator of so many aspects of immunity, immunologists have returned to undergraduate textbooks to uncover new metabolic‐focussed mechanisms which effect immunity. What has particularly captured the interest of so many are the various substrate preferences that different immune cells exhibit in order to generate ATP, and the differential ways in which immune cells utilise metabolic reprogramming to generate specific metabolites for pathogen defence, signalling, cytokine production and development. The number of different immune effector functions that are supported by distinct metabolic configurations is ever growing, so much so that metabolism is becoming as integral to immunity as the immune cells and mediators themselves (Figure 1).

**Figure 1:**
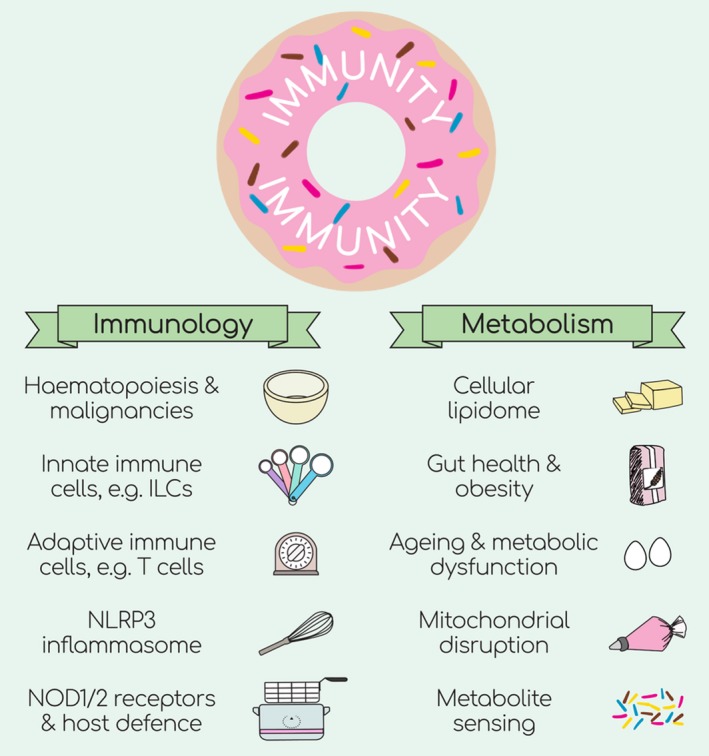
This Special Feature comprises five articles that detail different aspects of cellular metabolism that support immune cell function: the role of lipids in haematopoiesis; the interplay between type 2 immunity and gut homeostasis and obesity; the contribution of metabolic decline in ageing to T‐cell function; mitochondrial disruption and host defence; and bacterial metabolites and their sensing by NOD receptors. The articles detail how, together, immunology (i.e. tools) and metabolism (i.e. ingredients) combine to generate host immunity (i.e. donut).

One promise of immunometabolism research is that increased understanding of how pathways of cellular metabolism influence immune cell functions will open novel areas for the manipulation of immunity. This is a particularly promising avenue in metabolic demanding microenvironments such as the gut, infected lesions and tumors, where metabolic crosstalk between immune cells and commensals, pathogens or tumors is tightly linked to homeostasis and disease. Moreover, the influence of systemic factors such as ageing and the consumption of a ‘Western’ diet leading to conditions such as obesity add a further layer of complexity as these can influence local concentrations of metabolites, resulting in alterations in immune cell metabolic pathways and consequently effector functions. The challenge for all putative therapeutic interventions is to identify cellular metabolic mechanisms that are specific to immune cell functions.

This Special Feature of *Clinical & Translational Immunology* on *Regulation of Immunity and Infection by Pathways of Cellular Metabolism* comprises five publications that describe recent advances detailing metabolic mechanisms that support specific aspects of immunity. The articles encompass far‐reaching aspects of immunometabolism: from the role of lipids in the generation of immune cells during haematopoiesis; how innate and adaptive immune effector mechanisms are sensitive to altered metabolic states during obesity and ageing, respectively; to the specific metabolites and cellular metabolic mechanisms that support host responses to infections.

The cellular lipidome is composed of approximately 100 000 lipids. Best known for their role as major cellular fuel sources, lipids can also serve as signalling intermediates and provide structural integrity to cells. Pernes *et al.* 1 review the interaction between lipid metabolism and haematopoiesis, the process responsible for generating leucocytes. While it is well established that fatty acid oxidation is a major energy source for haematopoietic stem cell self‐renewal and progenitor cell differentiation, the review highlights other contributions of the lipid network with an emphasis on non‐oxidative lipid metabolism. Importantly, understanding of lipid metabolism during haematopoiesis has consequences for the development of therapeutic targets to treat haematological malignancies, including leukaemia.

Currently, it is estimated that there are more than 2 billion people worldwide who are overweight or obese. Type 2 immunity, which involves a complex array of immune cells including M2 macrophages, eosinophils, ILC2s, Tregs and iNKT cells, maintains an anti‐inflammatory milieu within adipose tissue. Obesity disrupts this status leading to inflammation and insulin resistance. In the review, Harris *et al.* 2 argue that helminth‐induced type 2 immunity helps promote the survival of the infected host, in part through its impact on adipose tissue. How helminth infection improves systemic metabolism presents a unique and exciting opportunity to identify novel approaches to combat the current obesity epidemic.

T‐cell responses in the elderly are often dysfunctional resulting in reduced vaccine efficacy and enhanced vulnerability to infection and cancer. T cells rely on metabolic pathways for their homeostasis and the ability to respond to signals delivered via the T‐cell receptor and/or cytokines. In this review, Quinn *et al.* 3 summarise how metabolic alterations manifest during ageing and the impact this has on T‐cell immunity. For those new to the field, the review begins with a concise overview of the fundamentals of T‐cell metabolism and its regulation during naive and memory T‐cell homeostasis and T‐cell activation. How ageing impacts T‐cell signalling is reviewed, including the shifts in metabolic profiles exhibited by age‐associated T‐cell subsets. The authors highlight how understanding of immunometabolism and how it impacts T‐cell immunity will enable the design of metabolic interventions to improve T‐cell outcomes in the elderly.

Mitochondria produce ATP that fuels cellular function. Mitochondrial DNA (mtDNA) originates from the mitochondrion’s bacterial ancestry, and its release from mitochondria is detected by pattern recognition receptors that trigger immunomodulatory mechanisms. Here, Schroeder *et al.* 4 review the PRR that sense mtDNA and how mitochondria impact PRR activity during infection and disease. The authors discuss the proposed mechanisms that would enable permeabilisation of two mitochondrial membranes that envelope mtDNA and pose hypotheses for mechanisms by which mtDNA may directly or indirectly drive NLRP3 signalling. This is important because mitochondrial disruption and NLRP3 inflammasome activity is implicated in several clinical contexts including cardiovascular and neurodegenerative disease.

Peptidoglycan (PG) is a glycopeptide polymer in the cell wall of nearly all species of both gram‐positive and gram‐negative bacteria. Degradation of PG by bacterial and host enzymes elicits metabolites that signal microbial immunity in the host. Sensors of PG metabolites are the cytosolic nucleotide‐binding oligomerisation domain (NOD) proteins, Nod 1 and 2. In this last review, Hang *et al.* 5 discuss PG metabolites and recent efforts to generate chemical derivatives to increase their clinical use by enhancing potency and bioavailability and reducing their associated side effects. How PG metabolites are used as adjuvants and in cancer treatment is also reviewed.

## Conflict of interest

The authors declare no conflict of interest.
